# Effects of Matrix pH on Spontaneous Transient Depolarization and Reactive Oxygen Species Production in Mitochondria

**DOI:** 10.3389/fcell.2021.692776

**Published:** 2021-06-30

**Authors:** Jannatul Aklima, Takumi Onojima, Sawako Kimura, Kanji Umiuchi, Takahiro Shibata, Yusho Kuraoka, Yoshiki Oie, Yoshiki Suganuma, Yoshihiro Ohta

**Affiliations:** ^1^Department of Biotechnology and Life Science, Tokyo University of Agriculture and Technology, Koganei, Japan; ^2^Department of Biochemistry and Molecular Biology, University of Chittagong, Chittagong, Bangladesh

**Keywords:** mitochondria, matrix pH, membrane potential, transient depolarization, reactive oxygen species

## Abstract

Reactive oxygen species (ROS) oxidize surrounding molecules and thus impair their functions. Since mitochondria are a major source of ROS, suppression of ROS overproduction in the mitochondria is important for cells. Spontaneous transient depolarization of individual mitochondria is a physiological phenomenon widely observed from plants to mammals. Mitochondrial uncoupling can reduce ROS production; therefore, it is conceivable that transient depolarization could reduce ROS production. However, transient depolarization has been observed with increased ROS production. Therefore, the exact contribution of transient depolarization to ROS production has not been elucidated. In this study, we examined how the spontaneous transient depolarization occurring in individual mitochondria affected ROS production. When the matrix pH increased after the addition of malate or exposure of the isolated mitochondria to a high-pH buffer, transient depolarization was stimulated. Similar stimulation by an increased matrix pH was also observed in the mitochondria in intact H9c2 cells. Modifying the mitochondrial membrane potential and matrix pH by adding K^+^ in the presence of valinomycin, a K^+^ ionophore, clarified that an increase in the matrix pH is a major cause of ROS generation. When we added ADP in the presence of oligomycin to suppress the transient depolarization without decreasing the matrix pH, we observed the suppression of mitochondrial respiration, increased matrix pH, and enhanced ROS production. Based on these results, we propose a model where spontaneous transient depolarization occurs during increased proton influx through proton channels opened by increased matrix pH, leading to the suppression of ROS production. This study improves our understanding of mitochondrial behavior.

## Introduction

Mitochondria are double membrane-bound organelles, with inner membranes consisting of cristae and boundary membranes, which run parallel to the outer membrane (Alberts et al., 2015; Wolf et al., 2019). The mitochondria provide most of the adenosine triphosphate (ATP) required for cell activities. The energy for ATP synthesis is stored in the form of the proton motive force (pmf) by proton pumps in the electron transfer chain (ETC), which translocate protons from the matrix to the intracristal space (Rieger et al., 2014; Plecitá-Hlavatá and Ježek, 2016). The pmf comprises the membrane potential (Δψ_m_) and pH gradient (ΔpH) across the inner membrane. Particularly, Δψ_m_ has been studied to elucidate how the mitochondria transport ions, substrates, and proteins (Komlódi et al., 2018; Zorova et al., 2018). Δψ_m_ is also used as an index of the mitochondrial state related to cellular activities (Chalmers and McCarron, 2008; Kostic et al., 2018; Wescott et al., 2019). Thus, the control and observation of Δψ_m_ provide important information on mitochondria and cells.

The mitochondria are also the center for the production of reactive oxygen species (ROS), which serve as signaling molecules at low concentrations but have considerably damaging effects on cells at high concentrations. Moreover, the mitochondria are sensitive to ROS (Turrens, 2003; Nickel et al., 2014) and increase ROS production when damaged (Yan et al., 2013; Zorov et al., 2014; Zandalinas and Mittler, 2018). Therefore, the mechanism by which the mitochondria maintain ROS production is crucial for cells. So far, several studies have been performed on the effects of pmf on ROS production (Korshunov et al., 1997; Vinogradov and Grivennikova, 2005; Selivanov et al., 2008; Rottenberg et al., 2009; Liu, 2010; Pryde and Hirst, 2011; Dröse and Brandt, 2012; Komlódi et al., 2018). Generally, when electron transfer is not inhibited, the decrease in pmf reduces ROS production because ROS are mainly produced as superoxides by electron leakage from the ETC to molecular oxygen (Pryde and Hirst, 2011). Further, the effects the pmf on ROS production have been intensively studied. The generation of pmf is associated with an increase in Δψ_m_ and ΔpH. The latter comprises an increase in matrix pH and a decrease in pH in the intracristal space (pH_ics_). During reverse electron transfer (RET) from ubiquinol to complex I in a pathological condition such as ischemia-reperfusion, Δψ_m_ (Komlódi et al., 2018) and matrix pH (Lambert and Brand, 2004; Selivanov et al., 2008) were observed to have a large influence on ROS production. During forward electron transfer (FET) from complex I to complex IV, i.e., physiological electron transfer, ROS production strongly depends on the increase in matrix pH (Selivanov et al., 2008).

Transient depolarization is a transient loss of Δψ_m_ observed in both isolated and intracellular mitochondria. Since transient depolarization considerably alters mitochondrial function for a short period, it has been intensively studied. Several mechanisms underlying this process have been elucidated (Buckman and Reynolds, 2001; Jacobson and Duchen, 2002; Aon et al., 2003; Vergun et al., 2003; Hattori et al., 2005; Azarias et al., 2008; Hu et al., 2008; Lee and Yoon, 2014; Jeong et al., 2017; Higashi et al., 2020). Among transient depolarization, spontaneous depolarization of individual mitochondria are physiological phenomena widely observed from plants to mammals (Wang et al., 2016). Thus far, spontaneous transient depolarization concomitant with an increase in matrix pH has been proposed (Hattori et al., 2005) and observed (Santo-Domingo et al., 2013; Breckwoldt et al., 2014). Moreover, a transient increase in ROS production is observed with spontaneous transient depolarization (Wang et al., 2008; Schwarzländer et al., 2012; Hou et al., 2014; Kuznetsov et al., 2017). However, the physiological role of transient depolarization and the underlying mechanisms remain to be clarified.

In experiments examining mitochondrial behavior, isolated mitochondria have an advantage over intracellular mitochondria because we can exactly control the environments surrounding the mitochondria. In the present study, we characterized spontaneous transient depolarization using isolated mitochondria and compared these characteristics with those of intracellular mitochondria. Further, we examined the effects of transient depolarization on ROS production in the mitochondria. A decrease in pmf is considered to reduce ROS production, but mitochondrial ROS production is increased during transient depolarization (Wang et al., 2008; Schwarzländer et al., 2012; Hou et al., 2014; Kuznetsov et al., 2017). Therefore, it is unclear whether transient depolarization reduces ROS production. The current study investigated the relationship between transient depolarization and ROS production, particularly considering the relationships among transient depolarization, matrix pH, and ROS production. This study provides valuable insights into the mechanism underlying the regulation of intracellular ROS levels, Δψ_m_, and matrix pH.

## Materials and Methods

### Isolation of Mitochondria

Mitochondria were isolated from porcine hearts by differential centrifugation with slight modification of previously described method (Palmer et al., 1977). Porcine hearts were purchased at a local slaughterhouse (Tokyo Meat Market). Animals were treated according to the guidelines of the Tokyo Meat Market and killed after anesthesia with carbon dioxide. The hearts of females or castrated males were removed from the bodies within 30 min after killing and kept on ice. The hearts were minced and incubated with 0.12 mg/mL subtilisin A (Sigma-Aldrich Japan, Japan) on ice at 4°C for 10 min in 10 mM Tris-HCl, 75 mM sucrose, 225 mM mannitol, and 0.5 mM ethylene glycol-bis (β-aminoethyl ether)-N,N,N′,N′-tetra acetic acid (EGTA), pH 7.4. Subtilisin A was removed by centrifugation at 1,000 *g* for 10 min, and the pellet was homogenized in the same buffer. The homogenate was centrifuged at 700 *g* for 10 min at 4°C. The supernatant was collected and further centrifuged at 4°C for 10 min at 5,000 *g*. The pellet was resuspended in Tris-HCl buffer (10 mM Tris-HCl, 250 mM sucrose, and 0.5 mM EGTA, pH 7.4) and kept on ice. One heart was used per mitochondria sample.

In all experiments, mitochondria were placed on ice before measurements and used for experiments within 4.5 h of preparation. Mitochondria were confirmed to be coupled with the respiratory control ratio of 4.1 ± 0.3 (*n* = 4) at 25°C. For this purpose, mitochondria were suspended at a concentration of 1 mg protein/mL in buffer containing 10 mM Tris-Hcl, 110 mM sucrose, 75 mM KCl, 1 mM MgCl_2_, 1 mM KH_2_PO_4_, and 1 mM malate (pH 7.4) at 25°C. To obtain state 3 respiration, 0.5 mM ADP was added. The protein content was determined using a protein assay with bovine serum albumin (Sigma-Aldrich Japan, Japan) as the standard. To adsorb mitochondria onto a glass-bottomed culture dish (35 mm diameter), a mitochondrial suspension (0.1 mg protein/mL) was placed in a culture dish and incubated at 4°C for 90 min (Shibata et al., 2019). The mitochondria were washed twice with the same buffer before microscopic measurements.

### Cell Culture

H9c2 rat cardiomyoblast cells were obtained from ATCC and were maintained in Dulbecco’s modified Eagle’s medium (Gibco, Life Technologies Corporation, United States) supplemented with 10% fetal bovine serum, 200 IU/mL penicillin, and 100 μg/mL streptomycin at 37°C in a humidified atmosphere of 5% CO_2_. The cells were cultured on glass-bottom culture dishes coated with collagen for 2–3 days before observation with an IX-70 microscope (Olympus, Tokyo, Japan).

### Fluorescence Imaging of Δψ_m_ in Mitochondria

For fluorescence imaging of Δψ_m_, isolated mitochondria were stained with tetramethylrhodamine ethyl ester (TMRE) (Thermo Fisher Scientific, United States), a potentiometric fluorescent dye (Duchen et al., 1998; Hüser and Blatter, 1999; Nakayama et al., 2002). Briefly, the isolated mitochondria were stained with 2 nM TMRE in Tris-HCl buffer for 10 min in the presence of 1 mg/mL BSA at 25°C (Hattori et al., 2005; Shibata et al., 2019). To measure the mitochondria in H9c2 cells, the cells were stained with 10 nM TMRE in HEPES-buffered saline (HBS; 10 mM HEPES, 120 mM NaCl, 4 mM KCl, 0.5 mM MgSO_4_, 1 mM NaH_2_PO_4_, 4 mM NaHCO_3_, 25 mM glucose, 1.2 mM CaCl_2_, and 0.1% BSA, pH 7.4) for 30 min at 37°C (Hirusaki et al., 2017). Then, the glass-bottom culture dish with isolated mitochondria or cells was placed on the stage of an inverted epifluorescence microscope (IX-70; Olympus; Tokyo, Japan). Measurements were performed using a 40 × (Uapo40 × /340, NA = 0.9; Olympus) or a 20 × objective lens (Uapo20 × /340, NA = 0.7; Olympus). TMRE was excited with 510 –550 nm light emitted from a 75 W xenon lamp. Emission >580 nm was measured using a cooled CCD camera (Sensicam QE, PCO AG; Kelheim, Germany). To estimate the time-resolved fluorescence, a series of image frames were acquired under computer control at 3-s intervals with 1 × 1 or 2 × 2 binning pixels. The exposure time for each frame was 1 s. During the remaining 2 s, the excitation light was cut off with a mechanical shutter to avoid mitochondrial damage possibly resulting from illumination. The intensity of illumination was also decreased to 25% with a neutral density filter to avoid photodynamic injury to mitochondria. The readout was digitized to 12 bits and analyzed with image-processing software (MetaMorph; Universal Imaging; Downingtown, PA). The fluorescence of isolated mitochondria was observed at 25°C because isolated mitochondria were unstable and gradually lost their membrane potential at 37°C and were not viable after 30-min incubation. Mitochondria in living cells were observed at 37°C.

To analyze individual isolated mitochondria, the fluorescence intensity was averaged over an area of 0.6 μm^2^ in each mitochondrion. The fluorescence intensity of TMRE in buffer was measured in the same field as mitochondria and at a position where the fluorescence intensity was not affected by mitochondrial TMRE. The ratio of average TMRE fluorescence intensity in the mitochondrion to that of the background was obtained as the TMRE signal dependent on Δψ_m_ (Loew et al., 1993; Nakayama et al., 2002) and is shown as the TMRE ratio. The transient depolarization of individual isolated mitochondria was detected based on the TMRE ratio, as described previously (Hattori et al., 2005). To analyze the changes in Δψ_m_ of individual intracellular mitochondria, time-lapse images of TMRE fluorescence in the cells were obtained for 90 s with a 40 × objective lens and were analyzed using MetaMorph. Transient depolarization was identified as a decrease greater than 15% of the average intensity in an area >1.0 μm^2^ within 3 s. To estimate the Δψ_m_ in a whole cell, the TMRE fluorescence was integrated over the entire cell (Hirusaki et al., 2017) and represented as the percentage of the integrated cellular fluorescence to the average of the integrated fluorescence of control cells (Cell TMRE). We selected cells that were not in contact with surrounding cells to obtain the whole-cell fluorescence and to calculate the integrated fluorescence intensity. The control cells were not treated with reagents.

### pH Measurements in Cells and Isolated Mitochondria

To measure the pH in cells and mitochondria, H9c2 cells and isolated mitochondria were stained with 2’,7’-Bis(carboxyethyl)-5,6-carboxyfluorescein (BCECF) (Thermo Fisher Scientific, United States), a fluorescent pH indicator (Komlódi et al., 2018). Procedures for observation of BCECF in living cells and isolated mitochondria were performed at 37 and 25°C, respectively. For staining, isolated mitochondria were loaded with BCECF-acetoxymethyl ester (AM) at 5 μM for 30 min in Tris-HCl buffer and washed twice with the same buffer. Cells were stained with 5 μM BCECF for 30 min in HBS and washed three times with HBS.

The BCECF-labeled mitochondria and cells were observed with the same fluorescence microscope used for TMRE. BCECF was illuminated with 390–420 nm (shorter wavelength) or 470–490 nm (longer wavelength). BCECF fluorescence was recorded between 515 and 550 nm. Fluorescence images of the dye illuminated with a shorter wavelength (F_S_) and a longer wavelength (F_L_) were obtained every 1 min and analyzed as integrated fluorescence, as described previously (Hirusaki et al., 2017).

To calibrate the pH from the (F_L_/F_S_) ratio, the fluorescence ratio (F_L_/F_S_) of individual mitochondria was measured at different pH values in the presence of 5 μM carbonyl cyanide m-chlorophenyl hydrazine (CCCP) (Sigma-Aldrich, Germany) to equilibrate the pH between the buffer solution and the mitochondrial matrix. Similarly, in H9c2 cells, 30 μM CCCP was used to equilibrate the pH between the buffer solution and the cytosol. Data were fitted with least-square fitting of (F_L_/F_S_) = (A + B × 10^(7– pH)^)/(C + 10^(7– pH)^) (Supplementary Figure 1), as reported previously (James-Kracke, 1992).

### Detection of ROS Production

ROS production in isolated mitochondria was measured with an Amplex Red Hydrogen Peroxide Assay Kit. Isolated mitochondria were suspended at 0.1 mg/mL in Tris-HCl buffer with 0.1 unit/mL horseradish peroxidase, 50 μM Amplex Red (Thermo Fisher Scientific, United States) at 25°C. Fluorescence was measured at 585 nm using a spectrofluorometer (JASCO, FP-6500) (Grivennikova et al., 2018). The fluorescence increase was monitored for 8 min after substrate addition. Mitochondrial ROS production was determined using hydrogen peroxide as a standard.

The H9c2 cells were stained with MitoSOX Red (Thermo Fisher Scientific, United States), an indicator of superoxide anions in mitochondria (Wang and Zou, 2018), to observe ROS production. Briefly, H9c2 cells were loaded with 2.5 μM MitoSOX Red for 10 min at 37°C in HBS. MitoSOX Red fluorescence was observed as described for TMRE.

### Measurement of O_2_ Consumption in Isolated Mitochondria and H9c2 Cells

Respiration in isolated mitochondria and H9c2 cells was measured with an oxygen electrode (SI782; Strathkelvin Instruments, Scotland) (Li and Graham, 2012). The water-jacketed chamber was maintained at 25 and 37°C for isolated mitochondria and H9c2 cells, respectively, and constantly stirred with a magnetic stirring bar to maintain a homogeneous sample. Prior to use, the electrode was calibrated with air-saturated water, assuming an O_2_ concentration of 7.13 μg/mL. Samples were placed in a gas-tight chamber that was sealed using a plastic plunger. Before respiration measurements, the isolated mitochondria were incubated with 1 μM oligomycin in the presence or absence of 5 mM ADP for 20 min in Tris-HCl buffer to completely block F_o_F_1_-ATPase activity before oxygen consumption measurements. Mitochondria were then added at 2.25 mg/mL to Tris-HCl buffer containing 1 μM oligomycin with or without 5 mM ADP in the chamber, followed by 5 mM malate and glutamate. The decrease of O_2_ concentration in the mitochondrial suspension was recorded using the Strathkelvin software recording system. The respiration rate in H9c2 cells was measured at 1.92 × 10^6^ cells/mL HBS. CCCP was added at 1 μM to the cell suspension. The same amount of DMSO was used as a control.

### Statistical Analysis

We averaged the data produced using mitochondria prepared from at least three independent samples for both isolated mitochondria and H9c2 cells. The results are expressed as the mean ± standard error of the mean (SEM). Data were analyzed by a two-tailed analysis of variance (ANOVA), followed by the Student-Newman-Keuls test. The difference was considered statistically significant at *p* < 0.05.

## Results

### Correlation Among Transient Depolarization, Δψ_m_, Matrix pH, and ROS Production in Isolated Mitochondria

**FIGURE 1:**
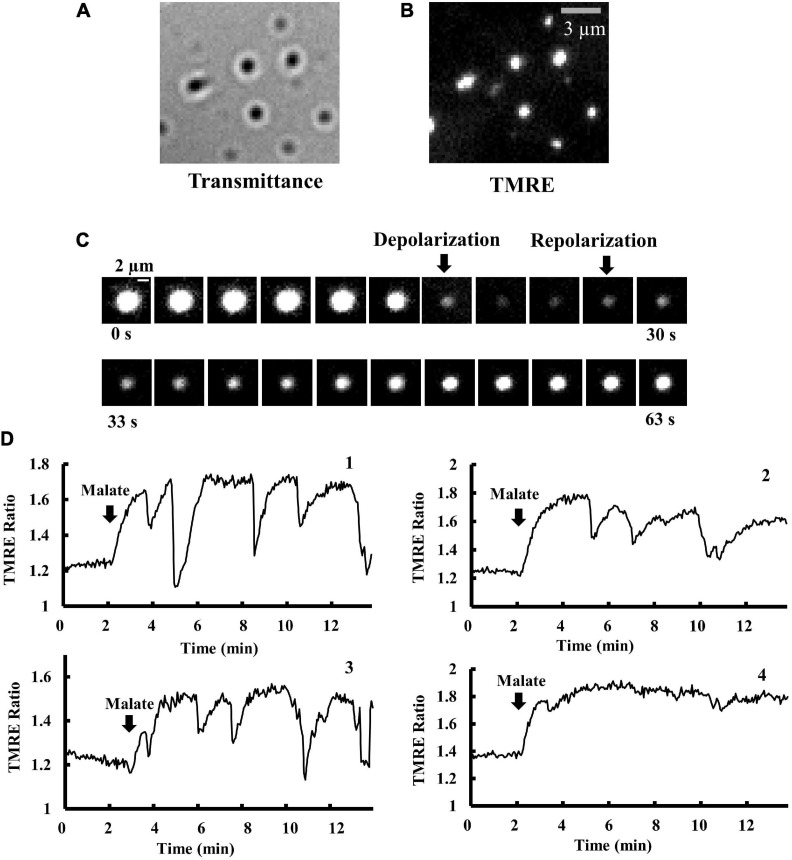
Time course of changes in Δψ_m_ in isolated mitochondria. **(A,B)** Optical images of isolated mitochondria adsorbed on a cover slip. **(A)** Brightfield and **(B)** fluorescence images of the same microscopic field as **(A)**. Bar, 3 μm. **(C)** Time-resolved fluorescence images of TMRE in a single mitochondrion. TMRE fluorescence was monitored in the presence of malate (5 mM). The arrows show the onset of depolarization and polarization. The time interval between images is 3 s. Bar, 2 μm. **(D)** Membrane potential (Δψ_m_) in a single mitochondrion in response to malate addition. The vertical axis represents the fluorescence intensity of TMRE in a single mitochondrion, normalized by TMRE fluorescence in the buffer (TMRE Ratio). At *t* = 2 min, 5 mM malate was added. The fluctuations of Δψ_m_ significantly depend on individual mitochondria. Δψ_m_ in mitochondria 1–3 is fluctuating, but not in mitochondrion 4.

Individual mitochondria were adsorbed onto a cover slip and observed using fluorescence microscopy. Individual isolated mitochondria were observed as small dots with a diameter of about 1 μm (Figures 1A,B). TMRE fluorescence in individual mitochondria was detected to monitor the time course of changes in Δψ_m_. TMRE fluorescence in mitochondria was normalized by background fluorescence (TMRE fluorescence in the medium) to obtain the Δψ_m_-dependent fluorescence intensity (Nakayama et al., 2002) and was shown as the TMRE ratio. An increase and a decrease in TMRE ratio indicate mitochondrial polarization and depolarization, respectively.

Isolated mitochondria showed spontaneous rapid depolarization and subsequent repolarization (Figure 1C). Before the addition of malate, the TMRE ratio was stable and less than 1.4 in mitochondria. Upon the addition of malate (5 mM), mitochondria became brighter (Figure 1D), indicating further polarization of the inner membrane. At the highly polarized state after the addition of malate, most mitochondria showed repeated cycles of rapid and transient depolarization followed by repolarization (Figure 1D). These oscillations were not synchronized among mitochondria, and the patterns of oscillations largely depended on individual mitochondria.

**FIGURE 2:**
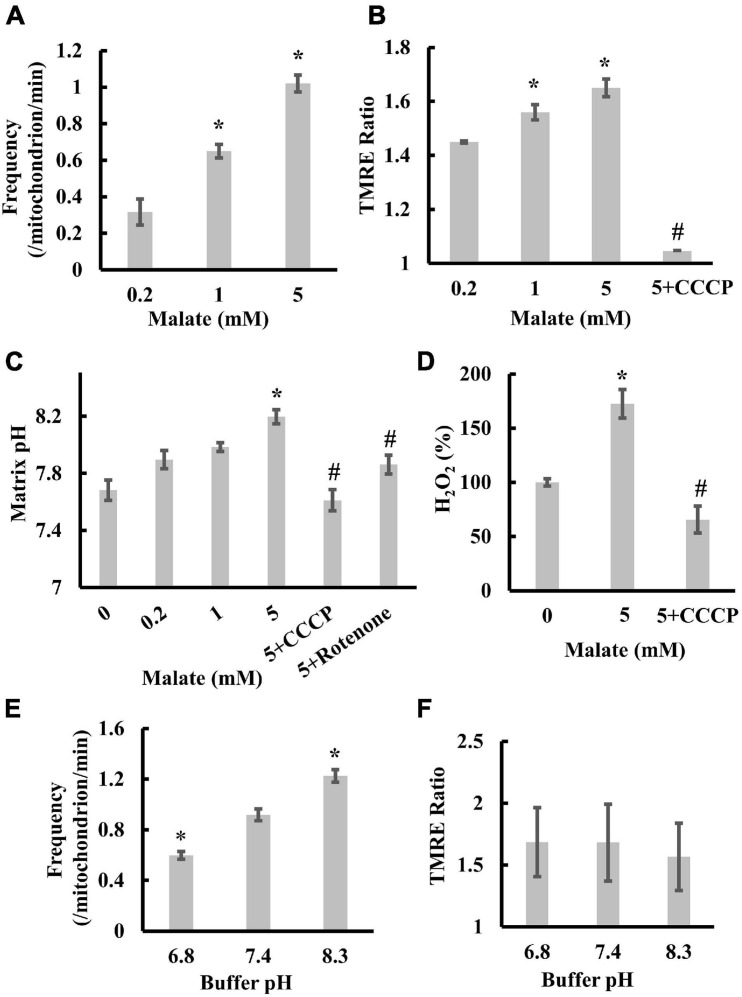
Correlation among transient depolarization, Δψ_m_, matrix pH, and ROS production in isolated mitochondria. **(A)** Effects of malate on the frequencies of transient depolarization. The frequency of depolarizations of the inner membrane is shown as the number of depolarizations/min/mitochondria. **(B)** The dependence of Δψ_m_ on malate and CCCP. The TMRE ratio was measured in individual mitochondria 1 min after the addition of malate. **(C)** Dependence of matrix pH on malate, CCCP, and rotenone. Matrix pH was measured in individual mitochondria 1 min after the addition of malate. **(D)** Effects of malate and CCCP on ROS production. H_2_O_2_ production in mitochondria without adding malate was set at 100%. **(E)** Effects of buffer pH on the frequencies of transient depolarization in the presence of 5 mM malate. **(F)** Dependence of Δψ_m_ on buffer pH. TMRE ratios for individual mitochondria were measured 1 min after the addition of 5 mM malate. Values represent the mean ± SEM (*n* > 50 for **A–C,E,F**; *n* = 3 for **D**). *p < 0.05 vs. 0.2 mM for **(A,B)**; vs. 0 mM for **(C,D)**; pH 7.4 for **(E)**. ^#^*p* < 0.05 vs. 5 mM for **(B,C,D)**.

To characterize transient depolarization, we first measured the frequencies of transient depolarization, the TMRE ratio, and the matrix pH in the presence of 0.2, 1, or 5 mM malate. With increasing substrate concentration, the frequency increased from 0.32 to 1.02/mitochondrion/min (Figure 2A). The TMRE ratio and matrix pH increased from 1.45 (0.2 mM) to 1.65 (5 mM) and from 7.68 (0 mM) to 8.19 (5 mM), respectively, after adding malate (Figures 2B,C). Moreover, rotenone, an inhibitor of complex I, significantly suppressed the malate-induced increase in TMRE ratio (Hattori et al., 2005) and matrix pH (Figure 2C), whereas the addition of CCCP significantly decreased the TMRE ratio and suppressed the increase in matrix pH and ROS production upon malate addition (Figures 2B–D). These results suggest a significant correlation among Δψ_m_, matrix pH, transient depolarization, and ROS production in mitochondria under these conditions. In addition, the increase in TMRE concentration from 2 to 200 nM, or the increase in excitation intensity, did not affect the frequency (Supplementary Figures 2A,B), suggesting that the transient depolarization was not due to a dye or illumination artifact.

Next, we examined the effects of pH on depolarization frequency. For this purpose, we replaced the pH 7.4 buffer with a buffer at an appropriate pH just before the experiments and measured TMRE fluorescence changes at pH 6.8, 7.4, and 8.3. The frequency significantly increased with increased buffer pH (Figure 2E). However, the increased buffer pH did not affect the mitochondrial Δψ_m_ (Figure 2F). Collectively, these results confirm that transient depolarization is enhanced by the increase in matrix pH but not by the polarization of the inner membrane because the increase in buffer pH increases the matrix pH (Zhang et al., 2018).

### Effects of Matrix pH on ROS Production in Isolated Mitochondria

**FIGURE 3:**
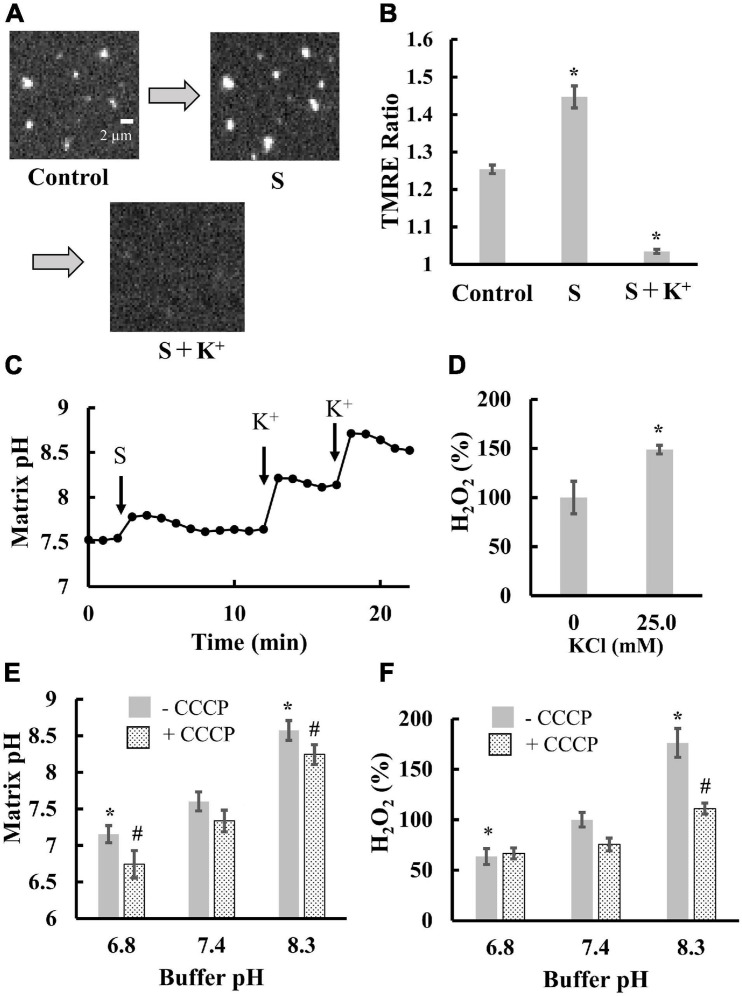
Effects of matrix pH on ROS production. Mitochondria isolated from porcine hearts were measured. Before measurements, mitochondria were incubated with 5 nM valinomycin for 10 min at 25°C. **(A)** Fluorescence images of TMRE in individual mitochondria. S, a mixture of malate and glutamate was added at a final concentration of 1 mM for both substrates, then 25 mM K^+^ was added. **(B)** Statistical analysis of TMRE ratio in individual mitochondria. S and K^+^ were added as described for **(A)**. Control, mitochondria before the addition of substrates. **(C)** Time course of changes in matrix pH in a single mitochondrion. At *t* = 3 min, S was added. At *t* = 12.5 and 17.5 min, K^+^ was added to mitochondria at a final concentration of 25 and 40 mM, respectively. **(D)** Effects of K^+^ on ROS production. ROS production was measured in the presence of malate and glutamate. The H_2_O_2_ production rate in the absence of K^+^ was set to 100%. **(E,F)** Effects of buffer pH on matrix pH **(E)** and ROS production **(F)**. Measurements were performed in the buffer with 1 mM malate and 1 mM glutamate. 5 μM CCCP was added. For **(F)**, the H_2_O_2_ production rate in the buffer at pH 7.4 (-CCCP) was set to 100%. Values represent the mean ± SEM (*n* > 50 for TMRE Ratio and matrix pH. *n* = 3 for ROS). *p < 0.05 vs. control for **(B)**; vs. 0 mM for **(D)**. In **(E,F)**, *p < 0.05 vs. pH 7.4 without CCCP, ^#^*p* < 0.05 vs. pH 7.4 with CCCP.

Next, we examined the contribution of matrix pH to ROS production. As ROS were generated in a pmf-dependent manner (Figure 2D), we attempted to distinguish between the effects of Δψ_m_ and matrix pH on ROS production. Thus, we sequentially added substrates (malate and glutamate) and K^+^ to mitochondria in the presence of valinomycin, a potassium ionophore. Addition of the substrates significantly polarized mitochondria (Figures 3A,B) and increased matrix pH (Figure 3C). Further K^+^ addition largely depolarized mitochondria (Figures 3A,B) and significantly increased matrix pH (Figure 3C). This could be due to the fact that in the presence of valinomycin, the influx of K^+^ in the medium into the matrix disrupted the proton pump barrier Δψ_m_, which stimulated proton outflow through the proton pump. Additionally, the depolarization of mitochondria and increased matrix pH by K^+^ significantly increased ROS production in mitochondria (Figure 3D). Therefore, these results suggest that the matrix pH has larger effects on ROS production than Δψ_m_. This conclusion was also supported by further experiments. The increase in the buffer pH from 6.8 to 8.3 did not affect Δψ_m_ (Figure 2F), whereas it increased the matrix pH significantly, regardless of the presence of CCCP (Figure 3E). Moreover, ROS production was also significantly increased with increasing buffer pH (Figure 3F). Although pH_ics_ decreases during proton pumping (Rieger et al., 2014), the decrease in pH_ics_ might not enhance ROS production because the decrease in the pH of the buffer around mitochondria decreased ROS production in mitochondria (Figure 3F).

Overall, in all cases tested, ROS production is synchronized with changes in matrix pH. Therefore, we concluded that matrix pH is the major regulator of ROS production in mitochondria.

### Effects of Transient Depolarization on Matrix pH, Electron Transfer and ROS Production in Isolated Mitochondria

In our previous study, we had shown that adenosine diphosphate (ADP) significantly suppressed transient depolarization in the presence of oligomycin (Uechi et al., 2006). Here, we examined the effects of ADP on matrix pH, oxygen consumption, and ROS production in the presence of oligomycin. First, oligomycin was added to inhibit the decrease in matrix pH by the entry of protons through F_o_F_1_-ATPase, which should affect transient depolarization and ROS production.

**FIGURE 4:**
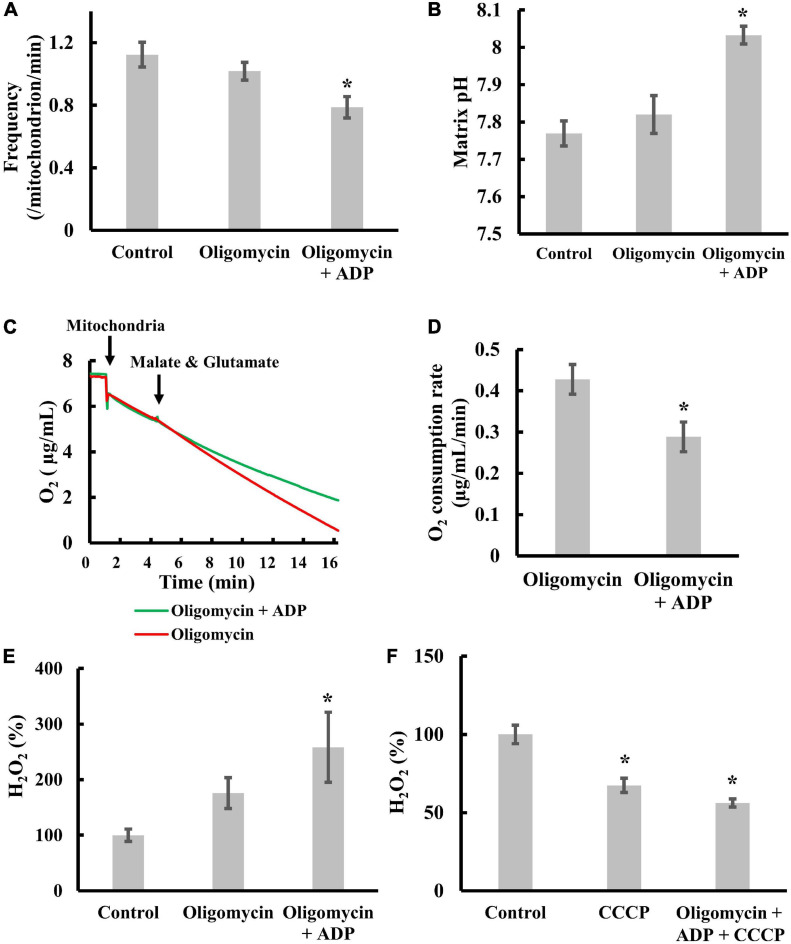
Effects of transient depolarization on matrix pH, electron transfer, and ROS production. Isolated mitochondria were measured in the presence of 5 mM malate and 5 mM glutamate. **(A–D)** Effects of ADP or oligomycin on **(A)** the frequency of the transient depolarizations, **(B)** the matrix pH, and **(C,D)** the respiration rate. Mitochondria were incubated for 20 min at 25°C with 5 mM ADP and 1 μM oligomycin before measurements. **(E)** Effects of ADP or oligomycin on ROS production. **(F)** Effects of CCCP on ROS production. The H_2_O_2_ production rate of control mitochondria was set to 100% for **(E)** and **(F)**. Values represent the mean ± SEM (*n* > 50 for frequency and matrix pH. *n* = 3 for respiration and ROS). *p < 0.05 vs. control without ADP and oligomycin for **(A,B,E,F)**; oligomycin for **(D)**.

We re-examined whether ADP significantly suppressed transient depolarization in the presence of oligomycin. As shown in Figure 4A, ADP significantly suppressed transient depolarization, as reported previously (Uechi et al., 2006). Since depolarization occurs upon the influx of cations into the matrix or the efflux of anions from the matrix, this result suggests that ADP suppresses cation influx or anion efflux.

Next, we examined whether ADP significantly increases matrix pH (Figure 4B) in the presence of oligomycin. The increase in matrix pH indicates either increased proton pumping or suppressed proton reentry. Therefore, to distinguish between these possibilities, we measured the effect of ADP on oxygen consumption in the presence of oligomycin. ADP significantly suppressed oxygen consumption (Figures 4C,D and Supplementary Figure 3A). Since oxygen consumption is coupled with proton pumping, this result indicates that proton pumping is not accelerated by ADP in the presence of oligomycin. Taken together, ADP suppresses the entry of protons into the matrix in the presence of oligomycin.

Third, we examined the effects of ADP on ROS production in the presence of oligomycin. When ADP was added to isolated mitochondria in the presence of oligomycin, we observed significantly enhanced ROS production (Figure 4E). In addition, CCCP inhibited this effect (Figure 4F). These results suggest that ADP enhanced-ROS production in mitochondria in the presence of oligomycin is counteracted by proton entry into the matrix. Since ROS are mainly produced as superoxides by electron leakage from the ETC to molecular oxygen (Duchen, 2004; Pryde and Hirst, 2011) when electron transfer via the ETC is inhibited, ADP does not suppress electron transfer in the presence of oligomycin when enough protons enter the matrix. ADP enhances ROS production by inhibiting electron transfer, which was further confirmed by the fact that ADP and oligomycin did not increase ROS production when electron transfer was fully inhibited with antimycin A (AA), a complex III inhibitor (Supplementary Figure 3B).

Collectively, addition of ADP in the presence of oligomycin suppressed the proton entry and increased matrix pH, resulting in ROS production. However, when transient depolarization was suppressed, we observed increased ROS production (Figure 4E). Therefore, the contribution of membrane potential to ROS production cannot be completely ruled out.

### Characterization of Transient Mitochondrial Depolarization in Cells

**FIGURE 5:**
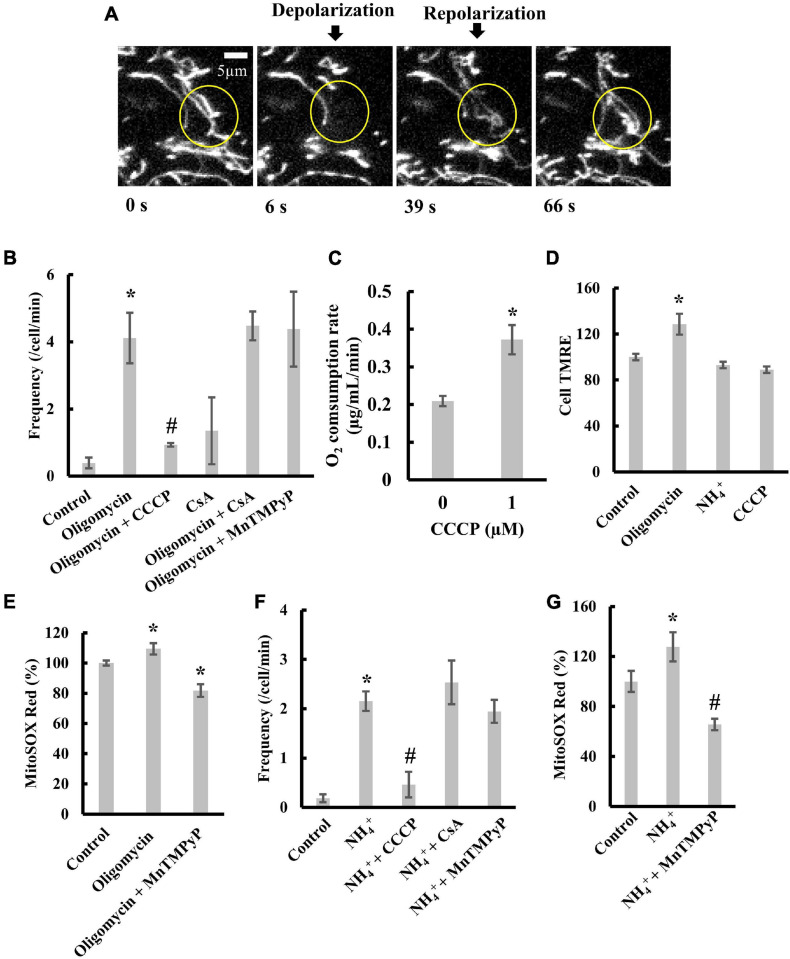
Transient depolarization of mitochondria in H9c2 cells. **(A)** Time-resolved fluorescence images of TMRE in an H9c2 cell. Mitochondria in the indicated circle were transiently depolarized. **(B)** Effects of oligomycin and CCCP on the frequency of transient depolarization. **(C)** Effects of CCCP on respiration. **(D)** TMRE fluorescence in H9c2 cells in the presence of oligomycin, NH_4_^+^, and CCCP. The fluorescence of TMRE in control cells was set to 100%. **(E)** Effects of oligomycin on ROS production by mitochondria in cells. Cells were incubated with 5 μM oligomycin at 37°C for 1 h before measurements. The fluorescence of MitoSOX in control cells was normalized to 100%. **(F,G)** Effects of NH_4_^+^ on **(F)** transient depolarization and **(G)** ROS production. Cells were incubated with 20 mM NH_4_^+^ at 37°C for 5 min before measurements. **(B–G)** CCCP, CsA, or MnTMPyP was added to cells, and they were incubated with 1 μM CCCP for 30 min, 2 μM CsA for 1 h, and 25 μM MnTMPyP for 1 h at 37°C before observation. Values represent the mean ± SEM (*n* > 10 for **B,D–G**, *n* = 3 for **C**). *p < 0.05 vs. control for **(B,D–G)**; 0 μM for **(C)**; ^#^*p* < 0.05 vs. oligomycin for **(B)**, and NH_4_^+^ for **(F,G)**.

The transient depolarization observed for isolated mitochondria or mitochondria in cells with permeable plasma membranes depends on the matrix pH (Hattori et al., 2005; Uechi et al., 2006). To examine the effects of matrix pH on transient depolarization in intact cells, we stained H9c2 rat cardiomyoblasts with TMRE. The time-lapse images of TMRE fluorescence in the cells showed that mitochondria in the indicated region were polarized at 0 s (Figure 5A). However, at 6 s, the mitochondria showed depolarization and repolarization at 39 s. Thus, mitochondrial depolarization and repolarization spontaneously occur in intact cells.

Oligomycin, an inhibitor of F_o_F_1_-ATPase, blocks the re-entry of protons into the matrix and thus increases pmf. In H9c2 cells, the addition of oligomycin largely increased the frequency of transient depolarization (Figure 5B). Further, the addition of CCCP (1 μM) significantly diminished the effect of oligomycin on depolarization frequency (Figure 5B). At this concentration, CCCP increased the respiration rate (Figure 5C), although it did not significantly decrease TMRE fluorescence of mitochondria in H9c2 cells (Cell TMRE) (Figure 5D and Supplementary Figure 4A). These results indicate that transient depolarization could be caused by an increase in pmf. Furthermore, adding cyclosporine A (CsA), an inhibitor of mitochondrial permeability transition (mPT), did not suppress the transient depolarization induced by oligomycin (Figure 5B). Manganese (III) tetrakis (1-methyl-4-pyridyl) porphyrin (MnTMPyP), a scavenger of superoxide anions, did not decrease the frequency of transient depolarization (Figure 5B). However, MnTMPyP decreased the amount of oligomycin induced superoxide anion in mitochondria (Figure 5E). These results indicate that oligomycin-induced transient depolarization is neither due to mPT nor ROS produced in mitochondria.

To further characterize transient depolarization, we added NH_4_^+^ to cells to increase the cytosolic pH (Supplementary Figure 4B; Yanaka et al., 1993; Bartolić et al., 2016), since increasing the pH of the solution around the mitochondria increases the matrix pH (Zhang et al., 2018). Treating cells with NH_4_^+^ stimulated transient depolarization without affecting Δψ_m_ (Figures 5D,F). The transient depolarization evoked by NH_4_^+^ was significantly suppressed by 1 μM CCCP (Figure 5F). Like transient depolarization induced by oligomycin, neither CsA nor MnTMPyP suppressed transient depolarization induced by NH_4_^+^ (Figure 5F). Moreover, MnTMPyP decreased ROS production (Figure 5G).

Taken together, these results suggest that the increase in matrix pH evokes transient depolarization in intact cells in a mitochondrial permeability transition- or a ROS-independent manner. These characteristics were similar to those observed for isolated mitochondria.

### Effects of H_2_O_2_ on Δψ_m_ and Transient Depolarization in H9c2 Cells

ROS did not affect transient depolarization under the above conditions. However, at high concentrations, ROS enhance (Buckman et al., 2001; Bajić et al., 2013) or destabilize Δψ_m_ (Satoh et al., 1997; Kuznetsov et al., 2017; Komlódi et al., 2018). Therefore, to understand how ROS affect Δψ_m_, we added H_2_O_2_ to cells and measured the behavior of Δψ_m_.

**FIGURE 6:**
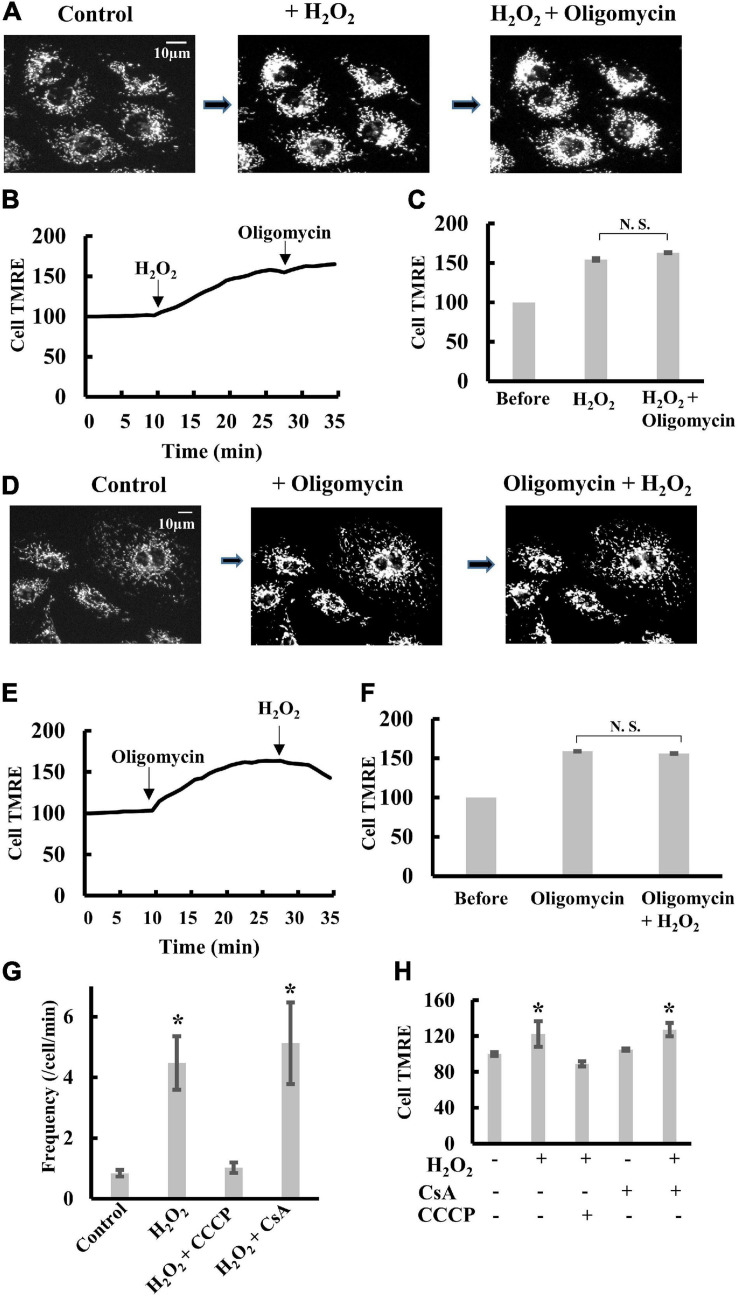
Effects of H_2_O_2_ on transient depolarization of mitochondria in H9c2 cells. **(A–C)** TMRE fluorescence in H9c2 cells upon sequential addition of H_2_O_2_ and oligomycin. H_2_O_2_ (1 mM) and oligomycin (5 μM) were added in order. **(A)** Fluorescence images of TMRE in H9c2 cells. **(B)** The time course of changes in Cell TMRE averaged over 10 H9c2 cells. Arrows indicate the addition of H_2_O_2_ or oligomycin. The fluorescence at t = 0 was normalized to 100%. **(C)** Cell TMRE before the addition of H_2_O_2_ and oligomycin was normalized to 100%. When H_2_O_2_ or oligomycin was present, cells were incubated with H_2_O_2_ or oligomycin for 20 min before measurements. **(D–F)** TMRE fluorescence in H9c2 cells upon sequential addition of oligomycin and H_2_O_2_. Oligomycin (5 μM) and H_2_O_2_ (1 mM) were added in order. **(D)** Fluorescence images of TMRE in H9c2 cells. **(E)** The time course of changes in Cell TMRE averaged over 10 H9c2 cells. Arrows indicate the addition of H_2_O_2_ or oligomycin. The fluorescence at t = 0 was normalized to 100%. **(F)** Cell TMRE before addition of H_2_O_2_ and oligomycin was normalized to 100%. When H_2_O_2_ or oligomycin was present, cells were incubated with H_2_O_2_ or oligomycin for 20 min before measurements. **(G)** Frequency of transient depolarization of mitochondria in H9c2 cells. The frequency is shown as the number of depolarizations/min/cells. CCCP and CsA were added as described in figure legends for Figure 3. When H_2_O_2_ was present, cells were incubated with H_2_O_2_ for 20 min before measurements. **(H)** Statistical analysis of Cell TMRE in H9c2 cells. H_2_O_2_, CCCP and CsA were added as described for **(G)**. Values represent the mean ± SEM (*n* > 10). *p < 0.05 vs. control for **(G)**; without CCCP, H_2_O_2_ and CsA for **(H)**.

The addition of 1 mM H_2_O_2_ to H9c2 cells induced significant polarization of mitochondria. Further addition of oligomycin did not significantly polarize mitochondria (Figures 6A–C and Supplementary Figure 5). Likewise, when oligomycin was added to cells before H_2_O_2_, H_2_O_2_ did not induce further polarization of mitochondria (Figures 6D–F). These results suggest that H_2_O_2_ inhibits F_o_F_1_-ATPase as reported previously (Terni et al., 2010; Qin et al., 2011; Wang et al., 2011). The frequency of transient depolarization was significantly increased by the presence of H_2_O_2_, and the increase was blocked by CCCP (Figure 6G), whereas CsA did not affect Δψ_m_ under the present conditions (Figure 6H). These results indicate that an increase in matrix pH evokes transient depolarization in intact cells in a mitochondrial permeability transition-independent manner because inhibition of F_o_F_1_-ATPase by H_2_O_2_ should cause an increase in matrix pH.

## Discussion

In this study, we demonstrated that (1) increased matrix pH stimulates spontaneous transient depolarization; (2) increased matrix pH also enhances mitochondrial ROS production, and 3) suppression of spontaneous transient depolarization without decreasing matrix pH suppresses respiration, elevates matrix pH, and enhances ROS production.

This study demonstrated that among Δψ_m_, matrix pH, and pH_ics_, an increase in matrix pH had the greatest effect on ROS production. These results are consistent with previous reports (Lambert and Brand, 2004; Selivanov et al., 2008; Komlódi et al., 2018). The enhancement of ROS production at a high matrix pH could be due to suppression of the conversion of semiquinone to ubiquinol, since semiquinone is converted to ubiquinol by receiving an electron and two protons. In the ETC, semiquinone donates electrons to oxygen molecules and is one of the major superoxide production sites (Selivanov et al., 2008; Komlódi et al., 2018). In this study, we examined the mechanism of ROS production by donating electrons to complex I to simplify the situation. However, in intracellular mitochondria, succinate also donates electrons to the ETC through complex II and induces RET in pathological conditions. Therefore, the use of succinate with substrates that donate electrons to complex I would help to understand ROS production mechanism in both physiological and pathological situations.

Our results indicate that increasing the matrix pH triggers ROS production and transient depolarization. This increase in matrix pH has been observed both *in vivo* and *in vitro* (Schwarzländer et al., 2012; Santo-Domingo et al., 2013; Wei-LaPierre et al., 2013; Breckwoldt et al., 2014). Importantly, increased matrix pH is transient, accompanied by the disappearance of Δψ_m_, and spontaneously occurs in individual mitochondria. Based on these observations, the elevation of matrix pH and depolarization of the inner membrane have been proposed as serial phenomena that occur within individual mitochondria (Schwarzländer et al., 2012). Furthermore, several studies have shown that the frequency of these phenomena depends on the pmf (Santo-Domingo et al., 2013; Wei-LaPierre et al., 2013; Feng et al., 2019) but not on the mPT (Santo-Domingo et al., 2013; Breckwoldt et al., 2014). Further, these phenomena are suppressed in acidic environments (Santo-Domingo et al., 2013; Wei-LaPierre et al., 2013). In this study, we showed that the frequency of transient depolarization depends on the pmf and increases under conditions where the matrix pH is high. We also showed that depolarization is independent of mPT. Taken together, the spontaneous transient depolarization we observed was essentially the same as the aforementioned depolarization, with a temporary increase in matrix pH.

**FIGURE 7:**
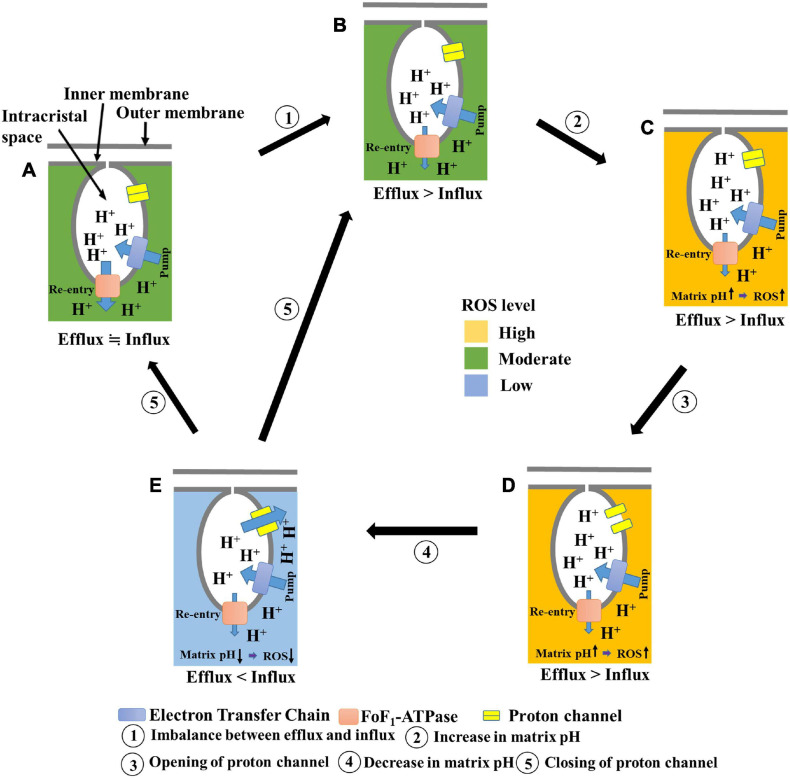
Schematic illustration of the proposed model for transient depolarization and ROS production. **(A)** Healthy mitochondria show proton efflux and influx equilibrium. ROS are moderately produced. **(B)** Influx of protons is suppressed. **(C)** Suppression of proton influx results in increased matrix pH. At this state, ROS production is enhanced. **(D)** The elevated matrix pH induces the opening of a proton channel. **(E)** Proton influx through the channel decreases pH. At this state, the level of ROS production is low. The proton channel is closed after the decrease in matrix pH.

In a previous study, we had proposed that elevated matrix pH induces transient depolarization of the mitochondria (Hattori et al., 2005; Uechi et al., 2006). In the present study, we found that elevated matrix pH also stimulates ROS production. In addition, we observed the following phenomena upon applying ADP to mitochondria in the presence of oligomycin: (1) suppression of cation influx or anion efflux through inhibition of transient depolarization; (2) suppression of proton influx into the matrix; and (3) increased ROS production through inhibition of proton influx. Based on these results, and by assuming that a significant number of protons enter the matrix during the transient depolarization, we have proposed a further developed model (Figure 7) in which an increase in matrix pH is linked to the dissipation of Δψ_m_ and ROS production. In this model, (1) ROS are more likely to be generated when the matrix pH increases due to an imbalance between proton influx and efflux (State A, B → State C); (2) the increased matrix pH induces the opening of a proton channel (State C → State D); (3) the resultant inflow of protons decreases the matrix pH and diminishes ROS production (State D → State E); and (4) following a decrease in matrix pH, the channel closes, and the pmf is formed (State E → State A, B).

This model is based on the principle that an increase in matrix pH induces depolarization, thus explaining why mitochondrial depolarization has been observed along with an increase in matrix pH in individual mitochondria (Schwarzländer et al., 2012; Santo-Domingo et al., 2013; Wei-LaPierre et al., 2013; Breckwoldt et al., 2014). Furthermore, it can be explained that the increase in matrix pH is transient, as protons flow back into the matrix during depolarization, which occurs after the increase in matrix pH. The aforementioned model can also explain how ROS increase the depolarization frequency (Schwarzländer et al., 2012; Breckwoldt et al., 2014). When ROS inhibit the re-entry of protons via F_o_F_1_-ATPase (Terni et al., 2010; Qin et al., 2011; Wang et al., 2011) rather than via proton pumps in complexes I, III, and IV under mild conditions, the elevation of ROS levels results in an increased matrix pH, which induces transient depolarization of the mitochondria. We observed mitochondrial polarization and stimulation of transient depolarization in the presence of H_2_O_2_, which supports the above interpretation. Thus, the elevation of ROS levels stimulates transient depolarization in living cells. However, when isolated mitochondria were measured in the absence of ADP, the depolarization frequency was not affected by H_2_O_2_, ROS scavengers (Hattori et al., 2005), or excitation light intensity. Overall, it can be inferred that ROS can induce transient depolarization via F_o_F_1_-ATPase inhibition rather than by directly inducing the opening of some cation channels. In addition, the model explains transient ROS production, called the superoxide flash (Wang et al., 2008; Schwarzländer et al., 2012; Hou et al., 2014; Kuznetsov et al., 2017), which is associated with a transient increase in matrix pH in a single mitochondrion because alkalization of the matrix promotes ROS production, and re-neutralization by proton influx suppresses it. Moreover, the time from depolarization to repolarization varies depending on the mitochondria. The transition of the proton channel from the open state to the closed state may involve mechanisms other than a simple pH-dependent proton attachment.

In this study, we could not provide direct evidence showing that the decrease in matrix pH occurs during the transient depolarization. Simultaneous detection of transient depolarization and the decrease in matrix pH in a single mitochondrion is necessary for proof of our model. Moreover, molecules that trigger the matrix pH-dependent transient depolarization, particularly the pH-sensor and proton conductive channels, have not been identified. Therefore, the frequency of intracellular mitochondrial depolarization could not be controlled, and the effect of matrix pH-dependent transient depolarization on mitochondrial ROS production in cells could not be detected. Identifying these molecules in future studies will further reveal the physiological role of pH-dependent transient depolarization as a mechanism for suppressing the production of ROS.

To date, mild uncoupling of mitochondrial oxidative phosphorylation is thought to play a role in suppressing ROS production and regulating bioenergetics (Korshunov et al., 1997; Cunha et al., 2011; Shabalina and Nedergaard, 2011; Vyssokikh et al., 2020). The transient depolarization observed in this study had a mild uncoupling property that suppressed ROS production. From this perspective, transient depolarization of the mitochondria may be considered a novel mechanism that modulates ROS production and bioenergetics in cells.

## Conclusion

This is the first study suggesting the relationship between spontaneous transient depolarization and ROS production. Spontaneous transient depolarization may decrease ROS production in the mitochondria by inhibiting sustained matrix pH elevation. Further studies on this mechanism are important because the overproduction of ROS can induce mitochondrial damage that impairs cellular function.

## Data Availability Statement

The original contributions presented in the study are included in the article/Supplementary Material, further inquiries can be directed to the corresponding author/s.

## Ethics Statement

We purchased porcine hearts at a local slaughterhouse (Tokyo Meat Market). The animals were killed for edible use, and the hearts were collected. The animals were treated and killed after anesthesia with carbon dioxide, according to the ethical guidelines of the Tokyo Meat Market.

## Author Contributions

JA, TO, SK, KU, TS, YK, YOi, and YS conducted the experiments and analyzed the results. JA and YOh designed the experiments and prepared the manuscript. All authors approved the final manuscript.

## Conflict of Interest

YOh was a co-inventor named on patent applications by LUCA Science Inc. The terms of this arrangement have been reviewed and approved by the Tokyo University of Agriculture and Technology, Japan, in accordance with its conflict-of-interest policies. The remaining authors declare that the research was conducted in the absence of any commercial or financial relationships that could be construed as a potential conflict of interest.

## Supplementary Material

Supplementary Figure 1 Calibration curve for determining matrix and cytosolic pH from BCECF fluorescence ratio. BCECF fluorescence was measured in the presence of 5 and 30 μM CCCP for isolated mitochondria and H9c2 cells, respectively, without the addition of malate. **(A)** Dots show experimental data. The solid line shows the theoretical curve of (F_L_/F_S_) = (A + B × 10^(7– *pH*)^)/ (C + 10^(7– *pH*)^) and is fitted to the experimental data with least-square fitting, where A is 0.045, B is 0.37, and C is 0.042. The dichroic mirror used is U-MWBV (Olympus, Japan). **(B)** In H9c2 cells, the above-mentioned theoretical curve is fitted to the experimental data with least-square fitting, where A is 0.567, B is 2.40, and C is 0.097. The dichroic mirror used is U-MNIBA (Olympus, Japan).

Supplementary Figure 2 Effects of TMRE concentration and illumination intensity on the transient depolarizations. Frequencies of the transient depolarization were determined in the presence of 1 mM malate. **(A)** Effects of TMRE concentration on the transient depolarization. **(B)** Effects of excitation intensity on the transient depolarization. Excitation intensity was changed with the neutral density filters with 12, 25, and 50% transmittance.

Supplementary Figure 3 Effects of ADP on respiration and antimycin A-induced ROS production. **(A)** Effects of ADP on respiration in the absence of oligomycin. ADP was added to the mitochondrial suspension at 5 mM. **(B)** Effects of ADP on antimycin A (AA)-induced ROS production in the presence of oligomycin. Mitochondria were incubated with 1 μM antimycin A for 20 min at 25°C. Values represent the mean ± SEM (*n* = 3). ^∗^*p* < 0.05 vs. control.

Supplementary Figure 4 Effects of CCCP and NH_4_^+^ on H9c2 cells. **(A)** Effects of CCCP concentration on Cell TMRE in H9c2 cells. **(B)** Effects of NH_4_^+^ on cytosolic pH in H9c2 cells. Values represent the mean ± SEM (*n* > 10). ^∗^*p* < 0.05 vs. 0 μM CCCP for **(A)**; control for **(B)**.

Supplementary Figure 5 TMRE fluorescence of mitochondria in H9c2 cells. The TMRE fluorescence image of H9c2 cells were obtained by using a 40 × (Uapo40 × /340, NA = 0.9; Olympus), with binning pixels 1 × 1, under computer control.
