# Supplementary material for: Effects of Matrix pH on Spontaneous Transient Depolarization and Reactive Oxygen Species Production in Mitochondria
Source: Front Cell Dev Biol. 2021 Jun 30;9:692776. doi: 10.3389/fcell.2021.692776 (PMC8278022; doi:10.3389/fcell.2021.692776)

## Supplementary Figure captions

**Figure S1. Calibration curve for determining matrix and cytosolic pH from BCECF fluorescence ratio.**

BCECF fluorescence was measured in the presence of 5 and 30 μM CCCP for isolated mitochondria and H9c2 cells, respectively, without the addition of malate. **(A)** Dots show experimental data. The solid line shows the theoretical curve of $(F_L/F_S) = (A + B \times 10^{(7\text{-pH})})/(C + 10^{(7\text{-pH})})$ and is fitted to the experimental data with least-square fitting, where A is 0.045, B is 0.37, and C is 0.042. The dichroic mirror used is U-MWBV (Olympus, Japan). **(B)** In H9c2 cells, the above-mentioned theoretical curve is fitted to the experimental data with least-square fitting, where A is 0.567, B is 2.40, and C is 0.097. The dichroic mirror used is U-MNIBA (Olympus, Japan).

**Figure S2. Effects of TMRE concentration and illumination intensity on the transient depolarizations.**

Frequencies of the transient depolarization were determined in the presence of 1 mM malate. **(A)** Effects of TMRE concentration on the transient depolarization. **(B)** Effects of excitation intensity on the transient depolarization. Excitation intensity was changed with the neutral density filters with 12, 25, and 50% transmittance.

**Figure S3. Effects of ADP on respiration and antimycin A-induced ROS production.**

**(A)** Effects of ADP on respiration in the absence of oligomycin. ADP was added to the mitochondrial suspension at 5 mM. **(B)** Effects of ADP on antimycin A (AA)-induced ROS production in the presence of oligomycin. Mitochondria were incubated with 1 μM antimycin A for 20 min at 25 °C. Values represent the mean ± SEM (n = 3). *$p < 0.05$ vs. control.

**Figure S4. Effects of CCCP and $NH_4^+$ on H9c2 cells.**

**(A)** Effects of CCCP concentration on Cell TMRE in H9c2 cells. **(B)** Effects of $NH_4^+$ on cytosolic pH in H9c2 cells. Values represent the mean ± SEM (n > 10). *$p<0.05$ vs 0 μM CCCP for A; control for B.

**Figure S5. TMRE fluorescence of mitochondria in H9c2 cells.**

The TMRE fluorescence image of H9c2 cells were obtained by using a 40× (Uapo40×/340, NA = 0.9; Olympus), with binning pixels 1 × 1, under computer control.

# Figure S1

A

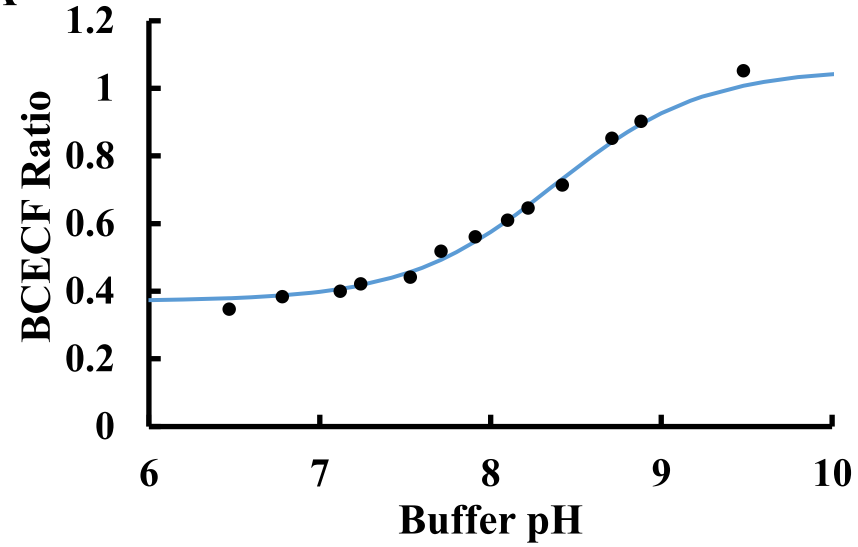

BCECF Ratio
1.2
1
0.8
0.6
0.4
0.2
0
6
7
8
9
10
Buffer pH


B

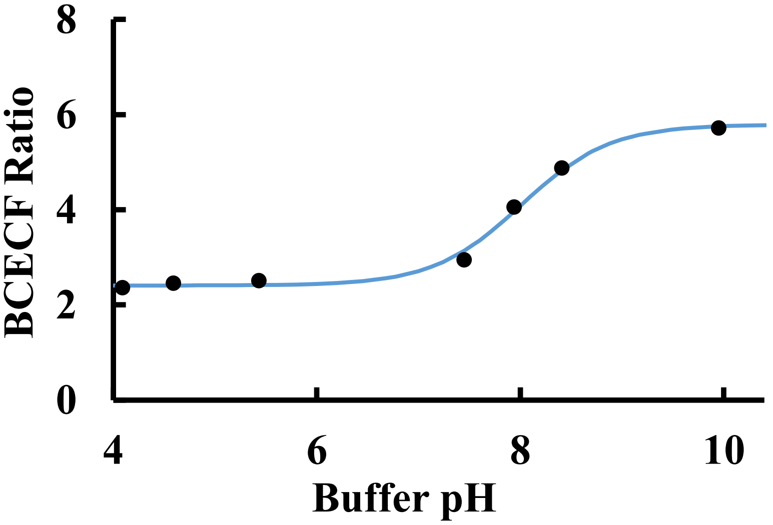

BCECF Ratio
8
6
4
2
0
4
6
8
10
Buffer pH

# Figure S2

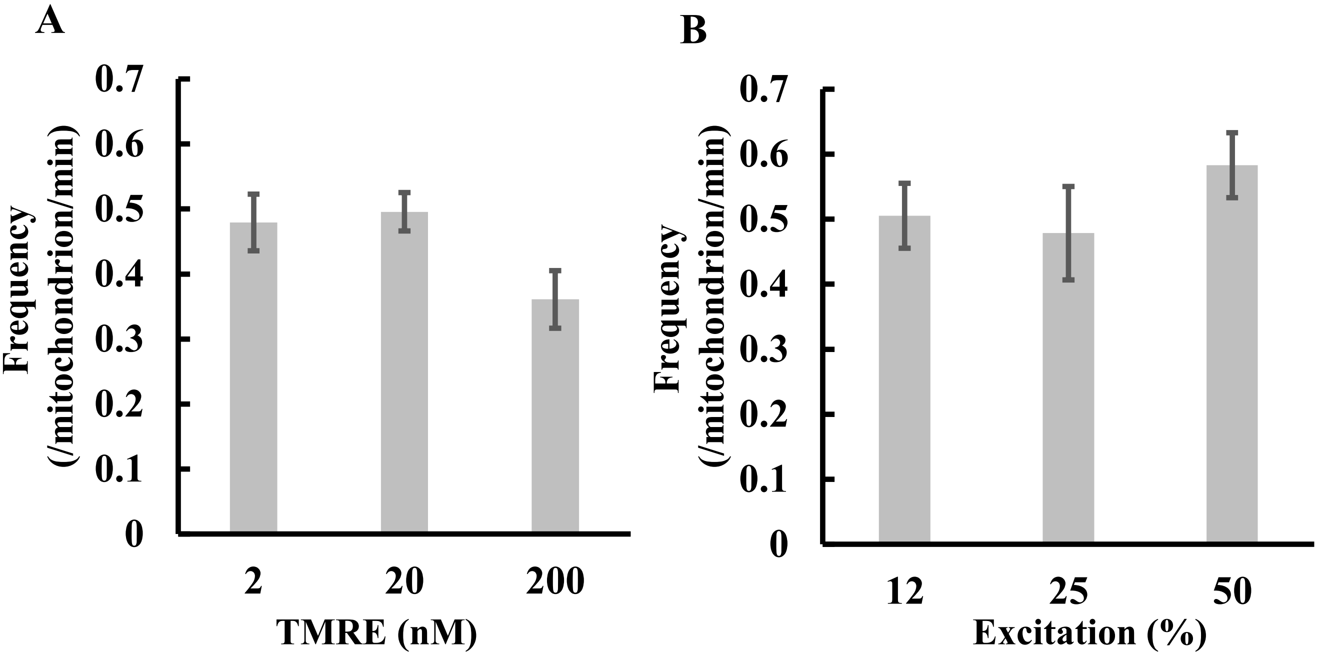
A
B
Frequency (/mitochondrion/min)
0
0.1
0.2
0.3
0.4
0.5
0.6
0.7
2
20
200
TMRE (nM)
12
25
50
Excitation (%)

# Figure S3

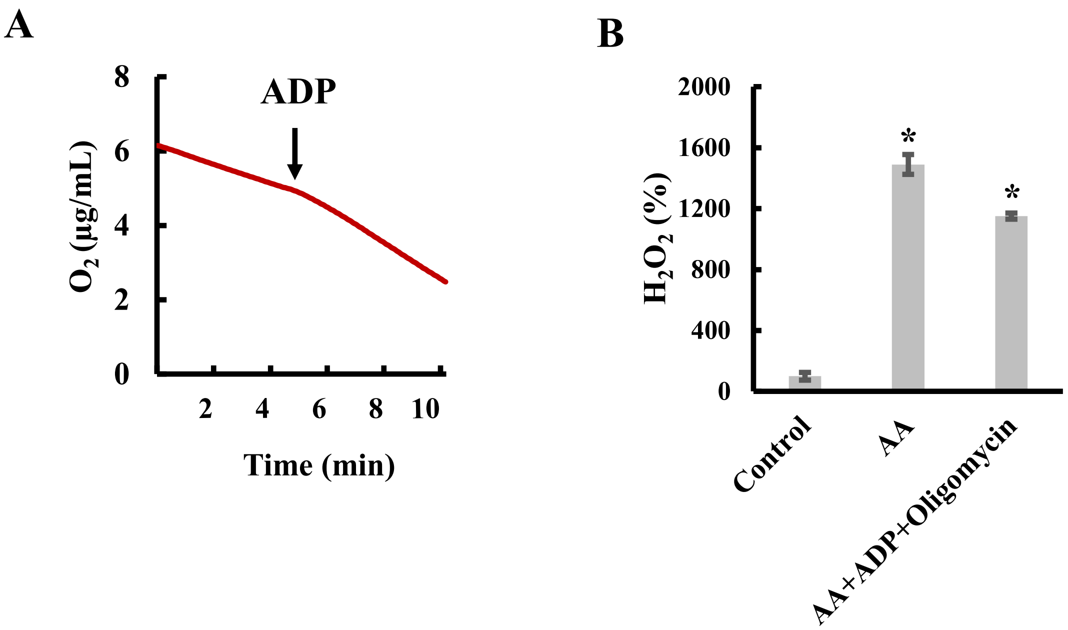
A
ADP
$O_2$ (µg/mL)
Time (min)
0
2
4
6
8
10
B
$H_2O_2$ (%)
0
400
800
1200
1600
2000
*
*
Control
AA
AA+ADP+Oligomycin

# Figure S4

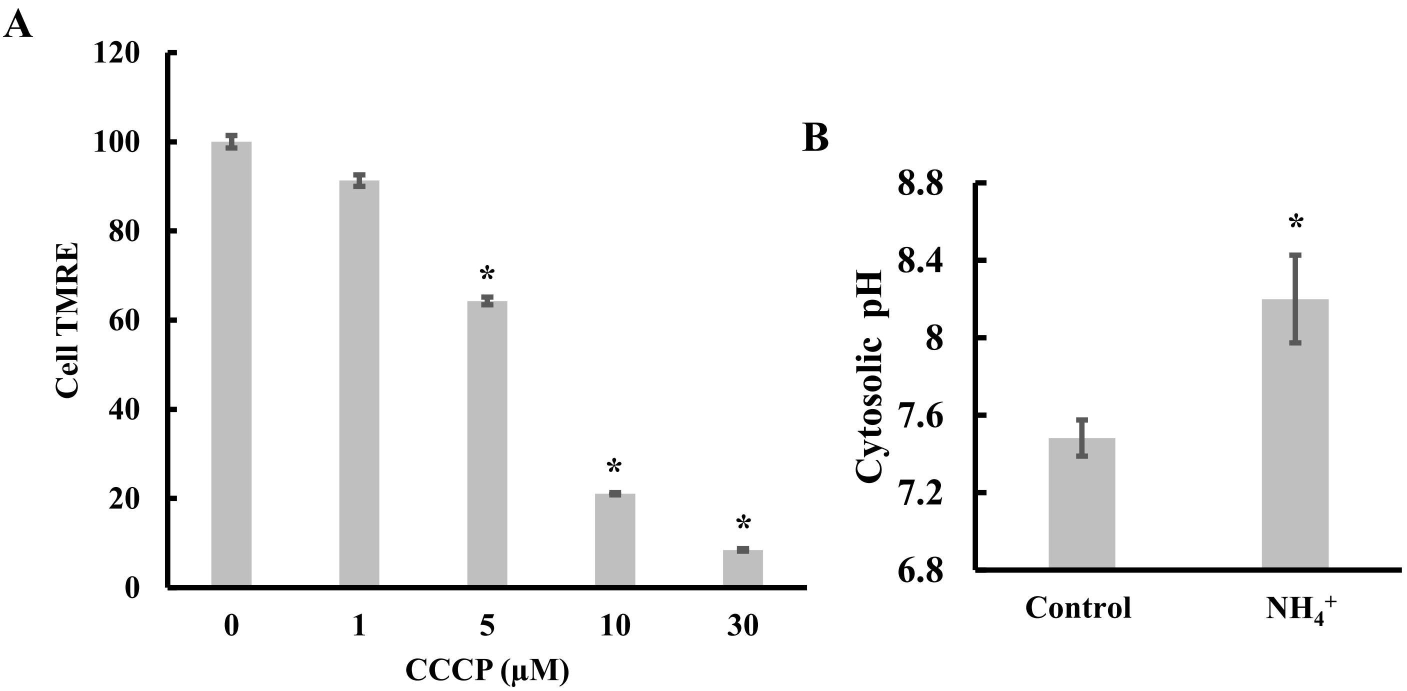
A
120
100
80
60
40
20
0
Cell TMRE
*
*
*
0
1
5
10
30
CCCP (µM)
B
8.8
8.4
8
7.6
7.2
6.8
Cytosolic pH
*
Control
$NH_4^+$

# Figure S5

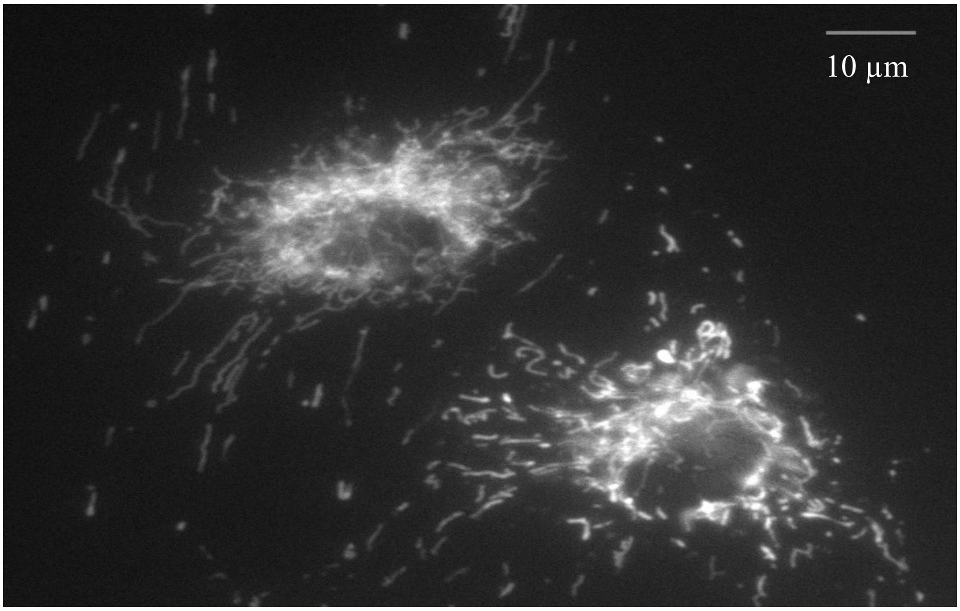

Supplement: Supplementary Figure 1 — Calibration curve for determining matrix and cytosolic pH from BCECF fluorescence ratio. BCECF fluorescence was measured in the presence of 5 and 30 μM CCCP for isolated mitochondria and H9c2 cells, respectively, without the addition of malate. (A) Dots show experimental data. The solid line shows the theoretical curve of (FL/FS) = (A + B × 10(7– pH))/ (C + 10(7– pH)) and is fitted to the experimental data with least-square fitting, where A is 0.045, B is 0.37, and C is 0.042. The dichroic mirror used is U-MWBV (Olympus, Japan). (B) In H9c2 cells, the above-mentioned theoretical curve is fitted to the experimental data with least-square fitting, where A is 0.567, B is 2.40, and C is 0.097. The dichroic mirror used is U-MNIBA (Olympus, Japan). [file Data_Sheet_1.pdf]
